# A Rare Presentation of Aggressive Renal Cell Carcinoma and the Utility of Early Molecular Testing in Rapidly Progressing Malignancies: A Case Report

**DOI:** 10.1093/oncolo/oyad280

**Published:** 2023-10-16

**Authors:** Daniel E McLoughlin, Nicholas Chevalier, Edwin Choy, Gregory M Cote, Xin Gao, Dejan Juric, Kerry L Reynolds

**Affiliations:** Division of Hematology and Oncology, Department of Medicine, Massachusetts General Hospital Cancer Center, Boston, MA, USA; Termeer Center for Targeted Therapies, Department of Medicine, Massachusetts General Hospital Cancer Center, Boston, MA, USA; Division of Hematology and Oncology, Department of Medicine, Massachusetts General Hospital Cancer Center, Boston, MA, USA; Termeer Center for Targeted Therapies, Department of Medicine, Massachusetts General Hospital Cancer Center, Boston, MA, USA; Division of Hematology and Oncology, Department of Medicine, Massachusetts General Hospital Cancer Center, Boston, MA, USA; Division of Hematology and Oncology, Department of Medicine, Massachusetts General Hospital Cancer Center, Boston, MA, USA; Termeer Center for Targeted Therapies, Department of Medicine, Massachusetts General Hospital Cancer Center, Boston, MA, USA; Division of Hematology and Oncology, Department of Medicine, Massachusetts General Hospital Cancer Center, Boston, MA, USA; Termeer Center for Targeted Therapies, Department of Medicine, Massachusetts General Hospital Cancer Center, Boston, MA, USA; Division of Hematology and Oncology, Department of Medicine, Massachusetts General Hospital Cancer Center, Boston, MA, USA; Termeer Center for Targeted Therapies, Department of Medicine, Massachusetts General Hospital Cancer Center, Boston, MA, USA; Division of Hematology and Oncology, Department of Medicine, Massachusetts General Hospital Cancer Center, Boston, MA, USA

**Keywords:** clear cell renal cell carcinoma, angiomyolipoma, precision medicine, exome sequencing, transcriptome

## Abstract

In rapidly progressing cancers, appropriate selection of first-line therapy is essential in prolonging survival. Alongside immunohistochemistry (IHC), comprehensive genomics, including whole exome and transcriptome sequencing (WES/WTS), can improve diagnostic accuracy and guide therapeutic management. Here, we report a young patient with rapidly progressing malignancy and unexpected post-mortem results, a scenario that may have been altered by early, comprehensive genomic sequencing. A 43-year-old man with no relevant medical history presented to the emergency department with progressive cough and dyspnea despite treatment for pneumonia. Radiology revealed enlarged subcarinal, hilar, retroperitoneal, and mesenteric lymph nodes, suspicious for metastasis, and a right kidney mass. Pathologic analysis of a retroperitoneal lymph node was felt to be most consistent with metastatic epithelioid angiomyolipoma (mEAML). Three weeks later, he was urgently treated with an mTOR inhibitor for presumed mEAML due to rapid clinical decline, and a subsequent 4R lymph node biopsy was performed to confirm the diagnosis and identify genomic targets via IHC and WES/WTS. Unfortunately, he developed hypoxic respiratory failure, and only posthumously did WES/WTS reveal pathogenic variants in BAP1 and VHL, consistent with clear cell renal cell carcinoma (ccRCC).

With an earlier ccRCC diagnosis, he would have received combination immunotherapy/tyrosine kinase inhibition, which has significantly greater activity than mTOR inhibition in ccRCC. He could have received systemic treatment earlier, with optimal therapy, while potentially carrying lower tumor burden and greater clinical stability. In cases of rapidly progressing malignancies with complex histopathological presentations, early comprehensive molecular-based testing can aid in diagnosis and critical therapeutic decision-making.

## Teaser

This case report shows that early comprehensive molecular-based testing can aid in diagnosis and critical therapeutic decision-making for patients with rapidly progressing malignancies with complex histopathological presentations.

**Key Points**

- Immunohistochemistry alone may not be sufficient for precise cancer diagnosis in all cases.
- In cases of rapidly progressing cancer with complex or unusual patterns of presentation, early molecular-based testing may be essential to confirm the diagnosis and select appropriate therapy.

## Background

Cancer is the second-leading cause of death in the US, with the American Cancer Society projecting over 600 000 new cancer deaths in 2022.^1^ In rapidly progressing malignancies with poor prognoses, an accurate diagnosis is essential when selecting first-line palliative therapies and prolonging survival. Despite advances in diagnostic pathology, modern-day immunohistochemistry (IHC) remains limited, with various cancers exhibiting shared or ambiguous histomorphological properties. IHC alone provides insufficient coverage of genomic variants, which necessitates further molecular profiling to accurately characterize tumors. Among the most comprehensive molecular profiling assays are whole exome sequencing (WES/DNA) and whole transcriptome sequencing (WTS/RNA), which read out the entirety of the expressed human genome within a cell. In a study of 200 patients with cancer of unknown primary (CUP), Ross et. al found that 96% of cases had an actionable mutation detected via comprehensive genomic profiling, and 85% of these were determined to be an alteration that could potentially guide decisions for targeted treatment.^2^

Here, we present a fulminant case of clear cell renal cell carcinoma (ccRCC) with an atypical pattern of presentation, highlighting the importance of pursuing early whole exome and transcriptome sequencing in the setting of rapidly advancing cancers with complex diagnoses.

## Patient Story

A 43-year-old man with no relevant medical history, who did not smoke and worked in insurance, presented to his local emergency department with cough and shortness of breath. He had been diagnosed with a diffuse bilateral pneumonia 1 week prior and was symptomatically worsening despite cefpodoxime, azithromycin, and prednisone. In the preceding weeks, he reported decreased appetite and oral intake, mild sinusitis, left lower extremity swelling, and purple discoloration of 2 toes.

Upon presentation to the emergency department, he was hypertensive (145/94), tachycardic (118 bpm), tachypneic (est. 20-25 breaths per minute), and hypoxic requiring supplemental oxygen (91% on 2 L, improved on 4 L). Laboratory testing was notable for hyponatremia (125 mmol/L), hypoalbuminemia (2.9 g/L), thrombocytopenia (121 10^9^/L), mild leukocytosis (11.3 K/µL), and hyperglycemia (314 mg/dL). His liver function tests and creatinine were within normal limits, and viral testing, including influenza and SARS-CoV-2, was negative.

**Figure 1.**
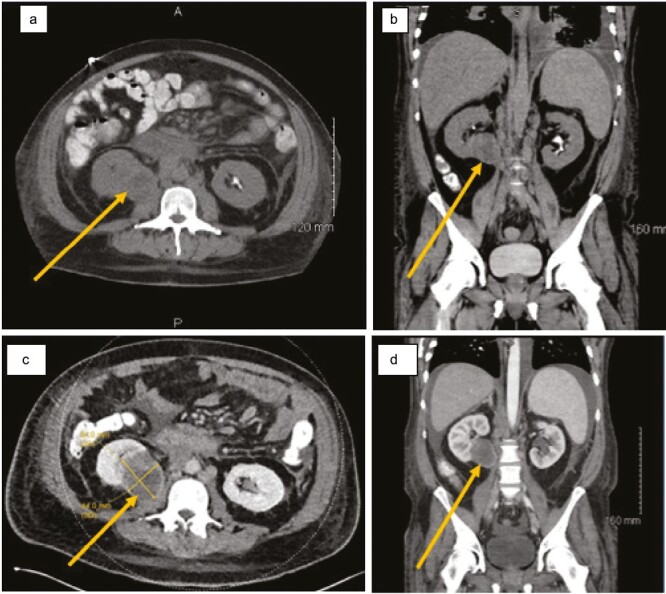
Computed tomography of right kidney mass at 2 timepoints. Axial (Panel **A**) and coronal (Panel **B**) CT at the time of initial workup demonstrated a mass in the right lower pole of the kidney, noted by the arrows, measuring 4.7 × 4.7 cm. Despite its considerable size, the mass was asymptomatic and, therefore, may have contributed to the challenge in diagnosing the cancer that was ultimately determined to be of renal origin by molecular testing. Axial (Panel **C**) and coronal (Panel **D**) CT approximately 3 weeks after his initial workup and diagnosis, with images taken just prior (coronal) and immediately following (axial) his transfer, demonstrated growth of the long axis of the right kidney mass to 6 cm, a rapid pattern of progression.

An initial chest x-ray showed worsening bilateral pneumonia and pleural effusion but could not rule out an underlying mass. Fluid from a subsequent thoracentesis demonstrated atypical epithelioid cells. Most notably, radiologic workup (CT) revealed a 4.7 × 4.7 cm mass in the lower pole of the right kidney (Fig. 1A and 1B), which was entirely asymptomatic, along with enlarged retroperitoneal and mesenteric lymph nodes and masses in infracarinal and hilar lymph nodes, suspicious for metastases.

A biopsy of a left retroperitoneal lymph node stained with synaptophysin (weakly), CD56, CAM 5.2, MNF 116, and MITF, and no significant staining was appreciated with chromogranin, HMB-45, S-100, Sox 10, cytokeratin AE1/AE3, CD3, CD20, PAX5, CD30, neurofilament, and CD15. Following interdepartmental consultation, although a ganglioneuroma was considered, the staining pattern, especially in the presence of a renal mass, seemed most consistent with epithelioid angiomyolipoma (eAML). A thoracentesis was performed for therapeutic and additional diagnostic purposes; however, cytology proved inconclusive. A consulting urologist noted that portions of the asymptomatic kidney mass likely contained fat cells, which would be consistent with angiomyolipoma, yet admitted that the diagnosis was “unusual.”

In approximately 3 weeks at his local hospital, his hypoxia progressively worsened, eventually requiring high-flow oxygen. Imaging demonstrated that the long axis of the renal mass had grown to 6 cm (Fig. 1C and 1D), and several of his lymphatic metastases had enlarged. Given the aggressive nature of his cancer and the complexity surrounding the diagnostic process, he was transferred to a large academic hospital for further workup and management. He was admitted with worsening thrombocytopenia (85 × 10^9^/L) and hypoxia requiring high flow oxygen up to 10 L. He was treated for a pulmonary infection with a broad-spectrum antibiotic regimen, including IV cefepime, IV vancomycin, oral azithromycin, and oral metronidazole. On the third day after his transfer, he suffered cardiac arrest, attributed to respiratory causes, from which he was resuscitated and subsequently intubated. With his clinical status worsening, the decision was made on the third day post-transfer to commence urgent systemic anticancer therapy; he was started on temsirolimus, an mTOR inhibitor, as first-line therapy for presumed metastatic eAML. Concurrently, the antibiotic regimen was narrowed to IV ceftriaxone when sputum culture resulted positive for *Klebsiella Pneumoniae*. Ceftriaxone was continued until completion of the course on day 8 post-transfer.

On day 5 post-transfer, a 4R lymph node was biopsied to confirm the diagnosis and identify genomic signatures for potential targeted therapy if mTOR inhibition proved inadequate. Tissue samples were sent to the pathology department for diagnosis and externally for whole exome and whole transcriptome sequencing.

In the following days, while tumor analysis was pending, he began to clinically decline, with his ventilation settings and thrombocytopenia worsening. Ultimately, 10 days after transfer, due to worsening clinical status, his goals of care were shifted to comfort measures only, and he was soon extubated. He died 1 month after his initial cancer diagnosis and underwent an autopsy the following morning.

**Figure 2.**
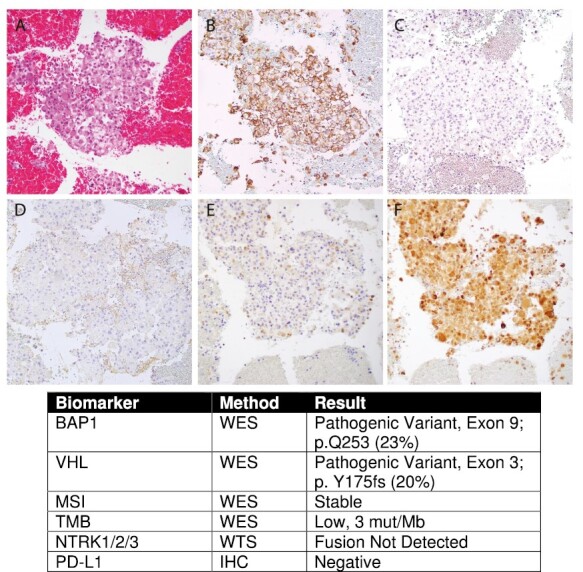
IHC staining and selected WES/WTS results from 4R lymph node. Hematoxylin and eosin stained sections of the 4R lymph node biopsy specimen showed malignant epithelioid cells with rhabdoid morphology, abundant eosinophilic cytoplasm, eccentrically placed nuclei, and prominent nucleoli (**A**). Lesional cells were positive for CA-IX (**B**) and PAX8 (**C**), negative for HMB45 (**D**), and retained expression of FH (**E**) and SDHB (**F**). Molecular testing demonstrated pathogenic variants in BAP1 and VHL.

The 4R lymph node pathology report, finalized after the patient’s passing, demonstrated a metastatic malignant epithelioid neoplasm with rhabdoid morphology; however, definitive classification of the tumor was deferred to autopsy. Meanwhile, the external molecular profiling report on the same sample resulted 12 days after the biopsy and 8 days post-mortem. Although the sample was collected and sent for sequencing with clinical intent, the patient had previously been consented via his healthcare proxy to IRB-approved protocol #13-416, under which the case could be carried to completion post-mortem for research purposes. The microscopic diagnosis was a malignant epithelioid neoplasm with clear cell and rhabdoid features, and molecular testing revealed pathogenic variants in BAP1 and VHL, resulting in a post-mortem diagnosis of metastatic clear cell renal cell carcinoma (mccRCC) (Fig. 2).

Post-mortem analysis confirmed these findings. Although partially necrotic, the renal mass and extensive metastases involving the retroperitoneum, thorax, and abdominal cavity were noted to have tumor cells with clear cytoplasm. The renal and lung blocks were positive for EMA and CA-IX. A cytology sample from his 4R lymph node biopsy was also reviewed at this time, from which lesional cells were positive for CA-IX and PAX8, with retained expression of FH and SDHB (Fig. 2). These results were most consistent with mccRCC with rhabdoid differentiation. His cause of death was confirmed to be mccRCC, which included lung metastases up to 1.2 cm, complicated by acute pneumonia.

## Molecular Tumor Board

Remarkably, the autopsy results provided a definitive diagnosis and categorically resolved the discrepancy between ccRCC and eAML. Unfortunately, the definitive diagnosis resulted after the patient’s death. Given the extensive, aggressive nature of his disease, rhabdoid differentiation, and persistent pneumonia-related complications despite appropriate antibiotic coverage, his clinical decline may have been unavoidable regardless of therapeutic intervention. However, given that his cancer not only directly caused fatal respiratory failure but also may have inhibited his ability to clear a likely hospital-acquired pulmonary infection, it bears asking how his clinical course may have been different had he had a clear diagnosis and received optimal systemic therapy weeks earlier.

Numerous studies have described the histologic and radiologic similarities between RCC and eAML,^3-5^ yet the management of these 2 cancers differs. While there is no consensus standard-of-care treatment for eAML,^6,7^ it has been reported that abnormal activation of the mTOR pathway may contribute to renal eAML growth and progression,^8^ and there have been reports of sustained responses to mTOR inhibition in angiomyolipomas.^9,10^ By contrast, although mTOR inhibitors are approved in RCC, they have limited activity, with an objective response rate (ORR) of 8.6%, median progression-free survival (PFS) of 3.8 months, and a modest overall survival (OS) benefit of 3.6 months (median OS 10.9 months) with first-line temsirolimus in patients with poor prognosis.^11^ In recent years, mTOR inhibition has been replaced by immune checkpoint inhibitors (ICI) or combination ICI and tyrosine kinase inhibitors (TKI) as first-line therapy for advanced RCC. Patients with mccRCC tend to respond significantly better to ICI/TKI combinations, with an ORR as high as 71%, a complete response rate as high as 16.1%, and a median PFS of 23.9 months with Pembrolizumab and Lenvatinib^12,13^

### What if molecular profiling had been performed at the time of the first biopsy?

This patient’s diagnosis was, at minimum, complex. The initial biopsy required interdepartmental consultation, a consulting urologist admitted the diagnosis to be “unusual,” and the presumed diagnosis was a cancer known to “mimic” another.^3-5,9^ Identifying molecular signatures was the key to confirming the diagnosis and selecting the most appropriate first-line therapy, and advanced molecular testing has demonstrated remarkable efficacy in predicting primary tumor of origin in CUP samples, with validation studies showing efficacy ranging from 75.7% (Zhao et al)^14^ to 94% (Abraham et al)^15^ to 98% accurate (Hainsworth et al).^16^ In addition, Ross et al (85% of 200 patients)^2^ and Cobain et al (80.5% of 1015)^17^ have found clinically relevant, potentially actionable mutations via molecular profiling in the vast majority of CUP patients.

When the 4R lymph node biopsy was performed, WES/WTS was finalized in 12 days. Had this been sought from the initial biopsy, the BAP1 and VHL mutations, nearly pathognomonic for RCC in the setting of a kidney mass,^18-21^ would have been discovered weeks earlier. Post-mortem analysis revealing PAX8 and CA-IX may have confirmed the RCC diagnosis,^22,23^ but molecular profiling identified markers that led to the same diagnosis in less than 2 weeks. This also underscores the imperative for future quality improvement efforts to reduce the turnaround time for genomic sequencing, as such cases necessitate results as quickly as possible.

For this patient, rapid, precise diagnosis of mccRCC would likely have been quickly followed by the commencement of ICI/TKI combination, which, in mccRCC, has an objective response rate that is 8 to 9 times higher, and a median progression-free survival that is over 6 times longer than temsirolimus.^11,12^ In addition, treatment would have commenced while he carried a lower tumor burden and a more stable hemodynamic and respiratory status. The potential impact of therapy would have been considerably higher. The stakes are now higher to get early and accurate diagnoses, as pursuing comprehensive molecular-based genomic testing has been shown to expand therapeutic options for patients with metastatic cancer,^24,25^ including those in clinical trials.^26^ Although comprehensive sequencing is still relatively new, evidence demonstrates that there may be trends toward better survival,^26^ and future research is needed to more definitively quantify this effect.

Genomic testing is not without its limitations. Whole exome and transcriptome sequencing does require more tissue than IHC alone, so having sufficient tumor is essential and may require additional cores to be taken at the time of biopsy. Although companies provide supplemental information regarding standard of care options and available clinical trials associated with biomarkers in their reports, it can be challenging to interpret complex genomic data and discern the difference between driver and passenger mutations. In addition, a single tumor site, which is often all that can be sampled during a routine core biopsy, may not capture the full complexity and heterogenous nature of the entire genomic landscape of a metastatic malignancy. However, outside the setting of autopsy, it is not feasible to sample every tumor in a widely metastatic cancer, so further investigation is needed to aid in selection of the most appropriate biopsy sites for comprehensive sequencing, or in non-invasive liquid biopsy options.

Cost and access to sequencing also merits discussion. In 2018, it was estimated that the price range for a single whole exome test fell between $555 and $5,169.^27^ Genomic testing panels are becoming increasingly utilized, with multiple commercially available platforms appearing on the market. It has been shown that a substantial number of patients with undiagnosed diseases and financial barriers to WES have actionable molecular diagnoses,^28^ underscoring the need for both expanded insurance coverage and the utilization of patient-assistance programs^29^ to increase access. In addition, advocacy efforts should be targeted at expanding access to WES/WTS to the most vulnerable and underserved communities. The cost-effectiveness of implementing next generation sequencing on a larger scale reveals limited, mixed evidence and remains an active of research; investigations have focused on specific populations ranging from treatment refractory cancers to the screening of healthy individuals.^30^ A study from Tan et al^31^ did find that the early use of more comprehensive genomic sequencing has been shown to be a potentially cost-effective option when compared to sequential testing in lung cancer patients in Asia. However, no study has yet analyzed the cost-effectiveness of early molecular profiling on patient outcomes among those with aggressive cancer, ambiguous diagnoses, and atypical clinical presentations, and this patient’s case demonstrates that there is, at minimum, a subset of patients who may derive significant clinical benefit from its utilization.

## Conclusion

In cases of rapidly progressing malignancies, unknown or unclear diagnoses, or both, pursuing comprehensive, molecular-based genomic sequencing as early as possible in the patient’s disease course may be essential to selecting the therapy that provides the best opportunity for clinical benefit.

## Data Availability

The data underlying this article will be shared on reasonable request to the corresponding author.

## References

[1] Siegel RL , Miller KD, Fuchs HE, Jemal A. Cancer statistics, 2022. CA Cancer J Clin. 2022;72(1):7-33. 10.3322/caac.21708 35020204

[2] Ross JS , Wang K, Gay L, et al. Comprehensive genomic profiling of carcinoma of unknown primary site: new routes to targeted therapies. JAMA Oncol. 2015;1(1):40-49. 10.1001/jamaoncol.2014.216 26182302

[3] Adanur S , Keskin E, Ziypak T, et al. Renal epithelioid angiomyolipoma mimicking urothelial carcinoma of the upper urinary tract. Arch Ital Urol Androl. 2014;86(3):235-236. 10.4081/aiua.2014.3.235 25308597

[4] Tsai CC , Wu WJ, Li CC, et al. Epithelioid angiomyolipoma of the kidney mimicking renal cell carcinoma: a clinicopathologic analysis of cases and literature review. Kaohsiung J Med Sci. 2009;25(3):133-140. 10.1016/s1607-551x(09)70052-x 19419918 PMC11918126

[5] Al Umairi R , Al Shamsi R, Kamona A, et al. Renal epithelioid angiomyolipoma: a case report and review of literature. Oman Med J. 2020;35(5):e178. 10.5001/omj.2020 33083036 PMC7568823

[6] Guo G , Gu L, Zhang X. Everolimus in invasive malignant renal epithelioid angiomyolipoma. Front Oncol. 2020;10:610858. 10.3389/fonc.2020.610858 33575217 PMC7870865

[7] Luo J , Liu B, Wang Y, et al. Comprehensive clinical and pathological analysis of aggressive renal epithelioid angiomyolipoma: report of three cases. Onco Targets Ther. 2014;7:823-827. 10.2147/OTT.S61524 24920923 PMC4043816

[8] Kenerson H , Folpe AL, Takayama TK, Yeung RS. Activation of the mTOR pathway in sporadic angiomyolipomas and other perivascular epithelioid cell neoplasms. Hum Pathol. 2007;38(9):1361-1371. 10.1016/j.humpath.2007.01.028 17521703 PMC2722219

[9] Tayal J , Doval DC, Kamboj M, Suryavanshi M. Case report of everolimus-induced sustained partial response in metastatic renal epithelioid angiomyolipoma. Turk J Urol. 2019;45(Supp. 1):S139-S142. 10.5152/tud.2018.66915 32027596 PMC6922043

[10] Bissler JJ , Kingswood JC, Radzikowska E, et al. Everolimus for angiomyolipoma associated with tuberous sclerosis complex or sporadic lymphangioleiomyomatosis (EXIST-2): a multicentre, randomised, double-blind, placebo-controlled trial. Lancet. 2013;381(9869):817-824. 10.1016/S0140-6736(12)61767-X 23312829

[11] Hudes G , Carducci M, Tomczak P, et al. Temsirolimus, interferon alfa, or both for advanced renal-cell carcinoma. N Engl J Med. 2007;356(22):2271-2281. 10.1056/NEJMoa066838 17538086

[12] Motzer R , Alekseev B, Rha SY, et al. Lenvatinib plus pembrolizumab or everolimus for advanced renal cell carcinoma. N Engl J Med. 2021;384(14):1289-1300. 10.1056/NEJMoa2035716 33616314

[13] Tran J , Ornstein MC. Clinical review on the management of metastatic renal cell carcinoma. JCO Oncol Pract. 2022;18(3):187-196. 10.1200/OP.21.00419 34529499

[14] Zhao Y , Pan Z, Namburi S, et al. CUP-AI-Dx: a tool for inferring cancer tissue of origin and molecular subtype using RNA gene-expression data and artificial intelligence. EBioMedicine. 2020;61:103030. 10.1016/j.ebiom.2020.103030 33039710 PMC7553237

[15] Abraham J , Heimberger AB, Marshall J, et al. Machine learning analysis using 77,044 genomic and transcriptomic profiles to accurately predict tumor type. Transl Oncol. 2021;14(3):101016. 10.1016/j.tranon.2021.101016 33465745 PMC7815805

[16] Hainsworth JD , Rubin MS, Spigel DR, et al. Molecular gene expression profiling to predict the tissue of origin and direct site-specific therapy in patients with carcinoma of unknown primary site: a prospective trial of the Sarah Cannon research institute. J Clin Oncol. 2013;31(2):217-223. 10.1200/JCO.2012.43.3755 23032625

[17] Cobain EF , Wu YM, Vats P, et al. Assessment of clinical benefit of integrative genomic profiling in advanced solid tumors. JAMA Oncol. 2021;7(4):525-533. 10.1001/jamaoncol.2020.7987 33630025 PMC7907987

[18] Dizman N , Philip EJ, Pal SK. Genomic profiling in renal cell carcinoma. Nat Rev Nephrol. 2020;16(8):435-451. 10.1038/s41581-020-0301-x 32561872

[19] Gallan AJ , Parilla M, Segal J, Ritterhouse L, Antic T. BAP1-mutated clear cell renal cell carcinoma. Am J Clin Pathol. 2021;155(5):718-728. 10.1093/ajcp/aqaa176 33210135

[20] Shuch B , Bratslavsky G, Linehan WM, Srinivasan R. Sarcomatoid renal cell carcinoma: a comprehensive review of the biology and current treatment strategies. Oncologist. 2012;17(1):46-54. 10.1634/theoncologist.2011-0227 22234634 PMC3267822

[21] Linehan WM , Ricketts CJ. The Cancer Genome Atlas of renal cell carcinoma: findings and clinical implications. Nat Rev Urol. 2019;16(9):539-552. 10.1038/s41585-019-0211-5 31278395

[22] Barr ML , Jilaveanu LB, Camp RL, et al. PAX-8 expression in renal tumours and distant sites: a useful marker of primary and metastatic renal cell carcinoma? J Clin Pathol. 2015;68(1):12-17. 10.1136/jclinpath-2014-202259 25315900 PMC4429054

[23] Courcier J , de la Taille A, Nourieh M, et al. Carbonic anhydrase IX in renal cell carcinoma, implications for disease management. Int J Mol Sci. 2020;21(19):7146. 10.3390/ijms21197146 32998233 PMC7582814

[24] Kawaji H , Kubo M, Yamashita N, et al. Comprehensive molecular profiling broadens treatment options for breast cancer patients. Cancer Med. 2021;10(2):529-539. 10.1002/cam4.3619 33274848 PMC7877356

[25] Ostrup O , Nysom K, Scheie D, et al. Importance of comprehensive molecular profiling for clinical outcome in children with recurrent cancer. Front Pediatr. 2018;6:114. 10.3389/fped.2018.00114 29732366 PMC5920151

[26] Coquerelle S , Darlington M, Michel M, Durand M, Borget I, Baffert S, Marino P, Perrier L, Durand-Zaleski I; NGSEco Group. Impact of next generation sequencing on clinical practice in oncology in France: better genetic profiles for patients improve access to experimental treatments. Value Health. 2020;23(7):898-906. 10.1016/j.jval.2020.03.005 32762992

[27] Schwarze K , Buchanan J, Taylor JC, Wordsworth S. Are whole-exome and whole-genome sequencing approaches cost-effective? A systematic review of the literature. Genet Med. 2018;20(10):1122-1130. 10.1038/gim.2017.247 29446766

[28] Reuter CM , Kohler JN, Bonner D, et al. Yield of whole exome sequencing in undiagnosed patients facing insurance coverage barriers to genetic testing. J Genet Couns. 2019;28(6):1107-1118. 10.1002/jgc4.1161 31478310 PMC6901723

[29] Chase L. What are patient assistance programs? 2022. http://www.goodrx.com/healthcare-access/patient-advocacy/what-are-patient-assistance-programs

[30] Tan O , Shrestha R, Cunich M, Schofield DJ. Application of next-generation sequencing to improve cancer management: a review of the clinical effectivenessand cost-effectiveness. Clin Genet. 2017;93(3):533-544. 10.1111/cge.13199 29265354

[31] Tan AC , Lai GGY, San G, et al. Utility of incorporating next-generation sequencing (NGS) in an Asian non-small cell lung cancer (NSCLC) population: Incremental yield of actionable alterations and cost-effectiveness analysis. Lung Cancer. 2020;139:207-215. 10.1016/j.lungcan.2019.11.022 31835042

